# Risk Factors and Mortality of COVID-19 in Patients With Lymphoma: A Multicenter Study

**DOI:** 10.1097/HS9.0000000000000538

**Published:** 2021-02-10

**Authors:** Isabel Regalado-Artamendi, Ana Jiménez-Ubieto, José Ángel Hernández-Rivas, Belén Navarro, Lucía Núñez, Concha Alaez, Raúl Córdoba, Francisco Javier Peñalver, Jimena Cannata, Pablo Estival, Keina Quiroz-Cervantes, Rosalía Riaza Grau, Alberto Velasco, Rafael Martos, Amalia Domingo-González, Laurentino Benito-Parra, Elvira Gómez-Sanz, Javier López-Jiménez, Arturo Matilla, María Regina Herraez, María José Penalva, Julio García-Suárez, José Luis Díez-Martín, Mariana Bastos-Oreiro

**Affiliations:** 1 Hospital General Universitario Gregorio Marañón, Madrid, Spain; 2 Hospital Universitario 12 de octubre, Madrid, Spain; 3 Hospital Universitario Infanta Leonor, Madrid, Spain; 4 Hospital Universitario Puerta de Hierro, Madrid, Spain; 5 Hospital Universitario Moncloa, Madrid, Spain; 6 Hospital Universitario Fundación Jiménez Díaz, Madrid, Spain; 7 Hospital Universitario Fundación Alcorcón, Madrid, Spain; 8 Hospital Universitario de La Princesa, Madrid, Spain; 9 Hospital Clínico San Carlos, Madrid, Spain; 10 Hospital Universitario de Móstoles, Madrid, Spain; 11 Hospital Universitario Severo Ochoa, Madrid, Spain; 12 Hospital Universitario Rey Juan Carlos, Madrid, Spain; 13 Hospital Universitario General de Villalba, Madrid, Spain; 14 Hospital Universitario de Getafe, Madrid, Spain; 15 Hospital Universitario del Sureste, Madrid, Spain; 16 Hospital Universitario Ramón y Cajal, Madrid, Spain; 17 Hospital Central de la Defensa Gómez Ulla, Madrid, Spain; 18 Hospital Universitario Infanta Sofía, Madrid, Spain; 19 Hospital Universitario Príncipe de Asturias, Madrid, Spain; 20 Instituto de Investigación Sanitaria Gregorio Marañón, Madrid, Spain.

## Abstract

Patients with cancer are poorly represented in coronavirus disease 2019 (COVID-19) series, and heterogeneous series concerning hematology patients have been published. This study aimed to analyze the impact of COVID-19 in patients with lymphoma. We present a multicenter retrospective study from 19 centers in Madrid, Spain, evaluating risk factors for mortality in adult patients with COVID-19 and lymphoma. About 177 patients (55.9% male) were included with a median follow-up of 27 days and a median age of 70 years. At the time of COVID-19 diagnosis, 49.7% of patients were on active treatment. The overall mortality rate was 34.5%. Age >70 years, confusion, urea concentration, respiratory rate, blood pressure, and age >65 score ≥2, heart disease, and chronic kidney disease were associated with higher mortality risk (*P* < 0.05). Active disease significantly increased the risk of death (hazard ratio, 2.43; 95% confidence interval, 1.23-4.77; *P* = 0.01). However, active treatment did not modify mortality risk and no differences were found between the different therapeutic regimens. The persistence of severe acute respiratory syndrome coronavirus 2-positive polymerase chain reaction after week 6 was significantly associated with mortality (54.5% versus 1.4%; *P* < 0.001). We confirm an increased mortality compared with the general population. In view of our results, any interruption or delay in the start of treatment should be questioned given that active treatment has not been demonstrated to increase mortality risk and that achieving disease remission could lead to better outcomes.

## Introduction

From December 2019, severe acute respiratory syndrome coronavirus 2 (SARS-CoV-2) has infected more than 70 million people worldwide, causing more than 1.5 million deaths. According to the Spanish Ministry of Health’s report, updated as of December 16, 2020, more than 1,773,000 cases and 48,000 deaths have been reported in Spain. More than 20% of cases have been registered in the Community of Madrid, which also accounts for more than 11,000 deaths, making it one of the most severely affected areas in Europe.

Patients with cancer are a particularly vulnerable population but only represent 0.5%-4% of the overall patients reported in the largest coronavirus disease 2019 (COVID-19) series.^1-6^ Specific studies focusing on patients with cancer have demonstrated higher incidences of severe outcomes and mortality rates compared with the general population.^3,7-10^ In these cancer series, hematology patients account for 20%-25% of the total,^3,7,9,11-15^ including a variable distribution of pathologies.

Heterogeneous series on COVID-19 in hematology patients have been published, reflecting mortality rates ranging from 30% to 40%^16-22^; however, these reports offer limited information on the characteristics of the various hematological diseases and the treatments used. A manuscript on COVID-19 and chronic lymphocytic leukemia^23^ reported a 33% mortality rate and interestingly found no relation between active treatment and mortality.

In this setting, we aimed to focus specifically on COVID-19 in patients with lymphoma, describing the epidemiology of the disease and analyzing predictors of poor outcomes.

## Methods

### Study design

This was a multicenter study performed in the Madrid area of Spain including 19 hospitals. The Madrid Haematology Association promoted the prospective collection of data on COVID-19 in hematology patients, including all types of hematological disorders.

From this initial registry, we focused on patients with lymphoma by retrospectively expanding the collected data with variables specific to this objective.

Inclusion criteria were adult patients with a previous or concomitant diagnosis of lymphoma and SARS-CoV-2 infection confirmed by reverse transcriptase-polymerase chain reaction (RT-PCR) in respiratory samples.

Cases were included from March 1, 2020, to May 30, 2020. The data cutoff was on July 15, 2020. Medical files were reviewed and data were incorporated into a standardized data collection form. All information was collected according to local data protection laws, and the study obtained the ethics committee’s approval.

The histologic diagnosis of the lymphoproliferative disorder and stage at diagnosis were recorded. Patients were grouped into 5 histologic categories: (1) diffuse large B-cell lymphoma (DLBCL); (2) follicular lymphoma (FL); (3) other aggressive lymphomas (including mantle cell lymphoma [MCL], primary effusion lymphoma [PEL], and natural killer [NK]-cell and T-cell neoplasms); (4) other indolent lymphomas (including marginal zone lymphoma [MZL], mucosa-associated lymphoid tissue [MALT] lymphoma, lymphoplasmacytic lymphoma [LPL], and hairy cell leukemia [HCL]); and (5) Hodgkin lymphoma (HL).

Risk stratification at lymphoma diagnosis was calculated according to histology-specific prognostic indexes (low risk: international prognostic index [IPI] 1-2, follicular lymphoma international prognostic index [FLIPI] 1-2, mantle international prognostic index [MIPI] low, and International Prognostic Score [IPS] Hasenclever 1-2; intermediate risk: IPI 3, FLIPI 3, MIPI intermediate, and IPS 3-4; high risk: IPI 4-5, FLIPI 4-5, MIPI high, and IPS 5-7).^24-27^ Patients were considered to be on active treatment if they had received treatment within the previous 3 months. The number of prior lines and response to treatment at the time of the COVID-19 diagnosis was also registered. In terms of therapeutic schemes, immunochemotherapy was divided into CD20-bendamustine and CD20-chemotherapy for regimens containing a monoclonal antibody targeting CD20 combined with chemotherapy. The CD20-chemotherapy category included regimens such as cyclophosphamide, doxorubicin, vincristine, and prednisone (R-CHOP); cyclophosphamide, vincristine, and prednisone (R-CVP); etoposide, methylprednisolone, high dose cytarabine, and cisplatin (R-ESHAP); or gemcitabine, and oxaliplatin (R-GemOx). The chemotherapy category included various combinations of chemotherapy agents; immunotherapy included patients on treatment with monoclonal antibody monotherapy (anti-CD20, anti-CD30) and checkpoint inhibitors (PD-1 inhibitors); targeted therapies included tyrosine kinase inhibitors. Lymphoma response at COVID-19 diagnosis was established based on Lugano’s response criteria.^28^ Patients with indolent lymphomas under observation were considered as patients with active disease.

The COVID-19 diagnosis was confirmed by positive SARS-CoV-2 RT-PCR (TaqMan 20119-nCoV Assay; Applied Biosystems).

Clinical, analytical, and radiological characteristics of COVID-19 presentation were collected. The confusion, urea concentration, respiratory rate, blood pressure, and age >65 (CURB-65)^29^ scale was used to measure the severity of the presentation in the absence of COVID-19-specific prediction tools at the time of data collection.

The date of COVID-19 diagnosis was defined as the day of the first positive RT-PCR for SARS-CoV-2. The date of the last positive and the first negative RT-PCR was recorded.

The primary end point of the study was to compare overall survival (OS) based on the presence of active treatment, active disease, and various therapeutic regimens. Another main objective was to infer mortality risk factors.

Secondarily, an analysis was performed for patients with persistently positive RT-PCR after 6 weeks from diagnosis. For this analysis, patients who died before the 6 weeks were excluded.

### Statistical analysis

Quantitative variables are expressed as the median and interquartile range (IQR). For the descriptive analysis, Pearson chi-square and Mann-Whitney *U* tests were used for comparing categorical and quantitative variables, respectively. Cox regression was used to infer mortality risk factors. For variables significantly related to mortality, a multivariate analysis was performed. The Kaplan-Meier method was used to estimate OS and log-rank tests were performed for the survival analysis. Statistical significance was defined by *P* < 0.05. We employed the SPSS statistics 25 software.

## Results

### Patient characteristics

Between March 1 and May 30, 196 cases of COVID-19 and lymphoma were recorded. Nineteen patients were excluded because of unconfirmed cases or lack of basic information. Ultimately, 177 patients (55.9% male) were analyzed, with a median follow-up of 27 days (9-49 d).

The median age was 70 years (IQR, 56-77 yr), and 72.9% of the patients had at least 1 comorbidity, with hypertension (41.2%), heart disease (19.2%), and diabetes (18.6%) being the most frequent. Table 1 summarizes demographic characteristics categorized by survival.

**Table 1:** Demographic Characteristics.

Characteristic	Entire Cohort (n = 177)	Alive (n = 116)	Dead (n = 61)
Age (median [IQR]), yr	70 (56-77)	64 (51-75)	75 (68.5-82)
<60 (n [%])	55 (31)	48 (41.4)	7 (11.5)
61-74 (n [%])	58 (32.8)	39 (33.6)	19 (31.2)
75-85 (n [%])	52 (29.4)	21 (18.1)	31 (50.8)
>85 (n [%])	12 (6.8)	8 (6.9)	4 (6.5)
Male (n [%])	99/177 (55.9)	65 (56)	34 (55.7)
Ethnic origin (n [%])			
White	153/168 (91.1)	96/109 (88.1)	57/59 (96.6)
Non-White	15/168 (8.9)	13/109 (11.9)	2/59 (3.4)
Comorbidities (median [IQR])	1 (0-2)	1 (0-2)	2 (1-3)
Comorbidities (n [%])			
None	51 (28.8)	42 (36.2)	9 (14.7)
1-2	94 (53.1)	57 (49.1)	37 (60.7)
3-4	26 (14.7)	14 (12.1)	12 (19.7)
>4	6 (3.4)	3 (2.6)	3 (4.9)
Types of comorbidity (n [%])			
Heart disease	34/177 (19.2)	13 (11.2)	21 (34.4)
Hypertension	73/177 (41.2)	41 (35.3)	32 (52.5)
Diabetes	33/177 (18.6)	20 (17.2)	13 (21.3)
Obesity	14/177 (7.9)	7 (6)	7 (11.5)
Dyslipidemia	27/177 (15.3)	18 (15.5)	9 (14.8)
Chronic pulmonary disease	23/177 (13)	13 (11.2)	10 (16)
Asthma	9/177 (5.1)	9 (7.8)	0 (0)
Chronic kidney disease	11/177 (6.2)	4 (3.4)	7 (11.5)
Chronic liver disease	4/177 (2.3)	2 (1.7)	2 (3.3)
Prior solid neoplasm	24/177 (13.6)	12 (10.3)	12 (19.7)
Previous treatments (n [%])			
ACE inhibitors	25/177 (14.1)	14 (12.1)	11/61 (18)
ARBs	17/177 (9.6)	8 (6.9)	9/61 (14.8)

ACE = angiotensin-converting enzyme; ARBs = angiotensin II receptor blockers; IQR = interquartile range.

### Lymphoma characteristics

The lymphoma subtype distribution was: 19 (10.75%) HL and 158 (89.3%) non-Hodgkin lymphoma (NHL). Of the NHL, 62 (35%) were FL, 39 (22%) DLBCL, 27 (15.3%) other aggressive lymphomas, and 30 (16.9%) other indolent lymphomas. The aggressive lymphomas included 13 MCLs, 12 T-cell lymphomas, 1 PEL, and 1 NK-cell lymphoma. Indolent lymphomas included 11 MZLs, 10 LPL, 5 HCLs, 3 MALTs, and 1 nonspecified indolent lymphoma.

Eighty-eight (49.7%) patients were on active treatment at the time of the COVID-19 diagnosis. See Table 2 for information related to previous treatments and lymphoma response at COVID-19 diagnosis categorized by histologic subtype.

**Table 2:** Lymphoma Characteristics.

Characteristic	DLBCL (n = 39)	Follicular Lymphoma (n = 62)	Hodgkin Lymphoma (n = 19)	Other Aggressive Lymphomas (n = 27)	Other Indolent Lymphomas (n = 30)
Median age (median [IQR])	70 (62-78)	68 (51-76)	58 (44-71)	72 (61-76)	76 (60-82)
Male (n [%])	26 (66.7)	29 (46.8)	12 (63.2)	16 (59.3)	16 (53.3)
Risk stratification^a^ (n [%])					
Low risk	6/33 (18.2)	18/55 (32.7)	3/15 (20)	4/21 (19)	10/19 (52.6)
Intermediate	10/33 (30.3)	17/55 (30.9)	8/15 (26.6)	7/21 (33.3)	7/19 (36.8)
High risk	17/33 (51.5)	20/55 (36.4)	4/15 (53.3)	10/21 (47.6)	2/19 (10.5)
Active treatment^b^	21/39 (53.8)	31/62 (50)	10/19 (52.6)	17/27 (63)	9/30 (30)
Lines of treatment (n [%])					
0	7/38 (18.4)	6/61 (9.8)	0/19 (0)	3/26 (11.5)	5/29 (17.2)
1	23/38 (60.5)	40/61 (65.6)	8/19 (42.1)	12/26 (46.2)	14/29 (48.3)
2	5/38 (13.2)	9/61 (14.8)	5/19 (26.3)	4/26 (15.4)	8/29 (27.6)
3	2/38 (5.3)	3/61 (4.9)	2/19 (10.5)	7/26 (26.9)	2/29 (6.9)
≥4	1/38 (2.6)	3/61 (4.9)	4/19 (21.1)	0/26 (0)	0/29 (0)
Therapeutics (n [%])					
CD20-chemotherapy^c^	27/33 (81.8)	22/54 (40.7)	0 (0)	5/22 (22.7)	4/24 (16.7)
CD20-bendamustine	2/33 (6.1)	7/54 (13)	0 (0)	6/22 (27.3)	5/24 (20.8)
Chemotherapy	4/33 (12.1)	4/54 (7.4)	12/19 (63.2)	8/22 (36.4)	5/24 (20.8)
Molecular targets	0 (0)	1/54 (1.9)	0 (0)	1/22 (4.5)	1/24 (4.2)
Immunotherapy^d^	0 (0)	20/54 (37)	7/19 (36.8)	2/22 (9.1)	9/24 (37.5)
Response^e^ (n [%])					
Complete response	14/38 (36.8)	34/61	12/19	12/27	11/29
Partial response	2/38 (5.3)	9/61	2/19	5/27	9/29
Progression	8/38 (21.1)	8/61	5/19	6/27	2/29
Not valuable	14/38 (36.8)	10/61	0/19	4/27	7/29
Active disease^f^ (n [%])	10/24 (41.7)	17/51 (33.3)	7/19 (36.8)	11/23 (47.8)	11/22 (50)
Admission (n [%])	35 (89.7)	51 (82.3)	14/18 (77.8)	24/26 (92.3)	27 (90)
ICU admission (n [%])					
Yes	4 (10.3)	5 (8.1)	4 (21.1)	1 (3.7)	2 (6.7)
Not needed	18 (46.2)	44 (71)	11 (57.9)	18 (66.7)	19 (63.3)
Dismissed	17 (43.6)	13 (21)	4 (21.1)	8 (29.6)	9 (30)
Death (n [%])	19 (48.7)	15 (24.2)	7 (36.8)	10 (37)	10 (33.3)

^a^Risk stratification at diagnosis.

^b^Lymphoma treatment within the 3 previous months.

^c^CD20-chemotherapy includes RCHOP, R-CVP, R-ESHAP, or R-GemOx.

^d^Anti-CD20: 31 patients; brentuximab: 3 patients; nivolumab: 4 patients.

^e^According to Lugano response criteria.

^f^Partial response or progression.

DLBCL = diffuse large B cell lymphoma; ICU = intensive care unit; IQR = interquartile range; R-CHOP = cyclophosphamide, doxorubicin, vincristine, and prednisone; R-CVP = cyclophosphamide, vincristine, prednisolone; R-ESHAP = etoposide, methylprednisolone, high dose cytarabine, and cisplatin; R-GemOx = gemcitabine and oxaliplatin.

### COVID-19 presentation

The median time from symptom onset to diagnosis was 5 days (2-7 d). The most common symptoms at presentation were fever (75.7%), cough (65.7%), dyspnea (49.7%), myalgia (25.9%), and diarrhea (20.6%). Supplementary Table S1 (http://links.lww.com/HS/A134) summarizes symptoms as well as radiological and analytical presentations categorized by outcomes.

Dyspnea at presentation was more frequent in the group of patients who died (65% versus 41.7%; *P* = 0.003). This group also showed a lower lymphocyte count (0.4 versus 0.7 cells/μL; *P* = 0.016), lower platelet count (159 versus 199 × 10^9^/L; *P* = 0.009), and lower hemoglobin level (11.6 versus 12.8 g/dL; *P* = 0.004), as well as higher levels of lactate dehydrogenase (LDH) (395 versus 292 U/L; *P* < 0.001) and C-reactive protein (CRP) (11.7 versus 5.3 mg/dL; *P* < 0.001).

CURB-65 score at admission was significantly higher in the group of patients who died with a higher proportion of patients scoring 2 points (30% versus 9.6%) and ≥3 points (38.3% versus 6.1%) (*P* < 0.001).

### COVID-19 management

One-hundred fifty-one (86.3%) patients required hospital admission, with a median admission duration of 12.5 days (IQR, 7-26 d). About 73% of the patients were hospitalized in COVID-19 areas.

Supplementary Table S2 (http://links.lww.com/HS/A134) compiles the information on supplemental oxygen requirements and antiviral and antiinflammatory treatments. The most commonly used drugs were hydroxychloroquine (88.1%), lopinavir-ritonavir (50.3%), and azithromycin (44.6%). Corticosteroids were administered to 87 (49.2%) patients and 51 (28.8%) received tocilizumab.

### Clinical evolution and outcomes

Sixteen (9%) patients were admitted to the intensive care unit (ICU), for a median of 14.5 days (IQR, 5-29 d), with 11 (68.8%) patients requiring invasive mechanical ventilation. The ICU mortality rate was 62.5%.

Fifty-one (28.8%) patients were not considered to be eligible for ICU admission due to age and/or preexisting comorbidities. Three (5.9%) of these patients survived despite not having access to intensive care; however, 94.1% of them died.

Supplementary Table S3 (http://links.lww.com/HS/A134) compares patients admitted to the ICU with those dismissed. Patients admitted to the ICU had a median age of 62 years (IQR, 49-68 yr), compared with a median of 77 years (IQR, 73-82 yr) of those considered noneligible. In the group of patients admitted to ICU (n = 16), 81.3% were in complete response of their lymphoma, whereas in the group of patients considered noneligible (n = 51), only 28% were in complete response.

### Predictors of death

The overall mortality rate was 34.5%. Table 3 summarizes the univariate analysis of death predictors. Regarding demographic variables, age and presence of comorbidities, specifically heart disease, hypertension, and chronic kidney disease, were found to increase mortality risk (*P* < 0.05). As for COVID-19 presentation, a CURB-65 score of ≥3 as well as lower platelet count, lower hemoglobin, elevated d-dimer, CRP >10 mg/dL, and LDH >300 U/L were associated with an increased risk of death (*P* < 0.05).

**Table 3:** Univariate Analysis for Predictors of Death.

Predictors	HR (95% CI)	*P *^a^
Demographic variables		
Age (yr)	1.048 (1.027-1.068)	**< 0.001**
Age ≥70 yr (reference <70)	3.396 (1.938-5.948)	**< 0.001**
Male (reference female)	1.074 (0.652-1.769)	0.778
Presence of comorbidities	2.255 (1.133-4.360)	**0.016**
Number of comorbidities	1.313 (1.115-1.547)	**0.001**
Heart disease	2.964 (1.730-5.080)	**< 0.001**
Hypertension	1.794 (1.087-2.961)	**0.022**
Diabetes	1.125 (0.603-2.102)	0.711
Chronic kidney disease	2.364 (1.039-5.378)	**0.040**
COVID-19 presentation		
CURB-65 ≥3 (reference 0-1)	6.99 (3.794-12.884)	**< 0.001**
Neutrophil count (×1000 cells/μL)	0.959 (0.881-1.045)	0.340
Neutrophil <1500 cells/μL	1.540 (0.860-2.759)	0.146
Lymphocyte count (×1000 cells/μL)	1.015 (0.992-1.039)	0.212
Lymphocyte count <500 cells/μL	1.526 (0.922-2.527)	0.100
Lymphocyte count <1000 cells/μL	1.712 (0.853-3.982)	0.607
Platelet count (×100 × 10^9^/L)	0.768 (0.610-0.966)	**0.024**
Platelet count <140 × 10^9^/L	1.764 (0.244-12.753)	0.574
Hemoglobin (g/dL)	0.897 (0.807-0.996)	**0.043**
C-reactive protein >10 mg/dL	2.123 (1.275-3.533)	**0.004**
Ferritin >1000 μg/L	1.363 (0.759-2.445)	0.300
LDH >300 U/L	1.793 (1.033-3.114)	**0.038**
d-dimer (×ULN)	1.257 (1.013-1.559)	**0.038**
Lymphoma characteristics		
High-risk lymphoma^b^ (reference low-risk)	2.756 (1.240-6.122)	**0.013**
DLBCL (reference FL)	2.663 (1.351-5.248)	**0.005**
Active treatment^c^	1.182 (0.714-1.956)	0.515
Active disease^d^ (reference CR)	2.232 (1.249-3.986)	**0.007**

*P* values marked in bold indicate statistically significant results (*P* < 0.05).

^a^Cox regression.

^b^High risk according to prognostic index at diagnosis.

^c^Lymphoma treatment within the 3 previous month.

^d^Partial response or progression.

CI = confidence interval; COVID-19 = coronavirus disease-2019; CR = complete response; CURB-65 = confusion, urea concentration, respiratory rate, blood pressure, and age >65; DLBCL = diffuse large B cell lymphoma; FL = follicular lymphoma; HR = hazard ratio; LDH = lactate dehydrogenase; ULN = upper limit of normal.

Lymphomas stratified with a high-risk score at diagnosis and those with active disease had a higher risk of death (hazard ratio [HR] 2.75; 95% confidence interval (CI), 1.24-6.12; *P* = 0.013 and HR, 2.43; 95% CI, 1.23-4.77; *P* = 0.01, respectively). However, active treatment and the number of prior lines did not make significant differences in mortality. DLBCL, compared with FL, had a higher COVID-19 mortality risk (HR 2.66 [95% CI, 1.35-5.247]; *P* = 0.0028).

Table 4 represents the results of the multivariate analysis. In this adjusted analysis, only age ≥70 years, heart disease, chronic kidney disease, and CURB-65 score ≥2 remained independent death predictors. Active disease also remained as a death predictor.

**Table 4:** Multivariate Analysis for Predictors of Death.

Predictors	HR (95% CI)	*P *^a^
Age >70 yr	3.700 (1.500-6.000)	**0.002**
Heart disease	2.738 (1.480-5.065)	**0.001**
Hypertension	1.308 (0.715-2.392)	0.383
Chronic kidney disease	3.710 (1.848-7.447)	**< 0.001**
CURB-65 score ≥2	5.963 (3.069-11.588)	**< 0.001**
Platelets <140 × 10^9^/L	0.998 (0.528-1.886)	0.996
Lymphocytes <1000 cells/μL	1.065 (0.536-2.115)	0.180
Hemoglobin <12 g/dL	1.341 (0.670-2.823)	0.439
High-risk lymphoma^b^ (vs low-risk)	1.256 (0.670-2.356)	0.476
DLBCL (vs FL)	1.623 (0.756-3.483)	0.213
Active treatment^c^	1.681 (0.382-7.407)	0.493
Active disease^d^ (vs CR)	2.770 (1.143-6.712)	**0.024**

*P* values marked in bold indicate statistically significant results (*P* < 0.05).

^a^Cox regression.

^b^High-risk according to prognostic index at diagnosis.

^c^Lymphoma treatment within the 3 previous months.

^d^Partial response or progression.

CI = confidence interval; CR = complete response; CURB-65 = confusion, urea concentration, respiratory rate, blood pressure, and age >65; DLBCL = diffuse large B cell lymphoma; FL = follicular lymphoma; HR = hazard ratio.

### SARS-CoV-2 RT-PCR after 6 weeks

Information was available for 92 (52%) of the patients alive at 6 weeks regarding SARS-CoV-2 RT-PCR status. Of these, 70/92 (76.1%) had a negative RT-PCR, whereas 22/92 (23.9%) were persistently positive.

Persistence of positive RT-PCR was not significantly associated with lymphoma subtype (*P* = 0.540), therapeutic regimen (*P* = 0.669), active treatment (*P* = 0.108), or active disease (*P* = 0.392). We compared the proportion of patients who had received monoclonal antibodies targeting CD20 in the previous 12 months and found no differences between the negative RT-PCR group and persistently positive (30/70 versus 11/22; *P* = 0.385).

We found significant differences regarding mortality, with a 54.5% mortality rate in the persistently positive RT-PCR group compared with 1.4% in patients with a negative RT-PCR (*P* < 0.001).

At the time of the data analysis, 61 (34.5%) patients had died, 90 (50.8%) had been discharged and remained alive, and 26 (14.7%) remained hospitalized. Excluding these 26 and considering only the 151 patients who had died or been discharged, the mortality rate would be 40.4% (61/151).

The median age of the deceased patients was 75 years (IQR, 68.5-82 yr). (See Supplementary Table S4, http://links.lww.com/HS/A134).

**Figure 1.**
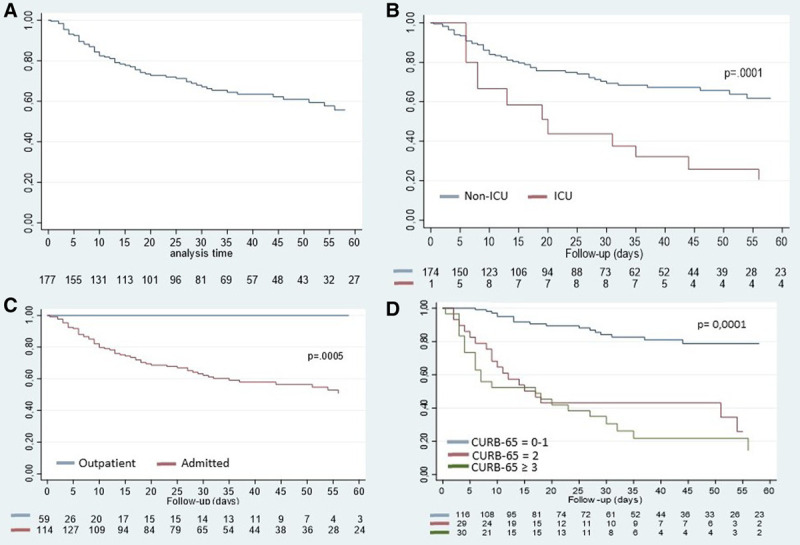
**OS results of the entire cohort and based on admission, ICU admission and CURB-65 score.** (A), Global OS. (B), OS comparing outpatients with inpatients. (C), OS for patients admitted in ICU vs no ICU. (D), OS categorized by CURB-65 score. CURB-65 = confusion, urea concentration, respiratory rate, blood pressure, and age >65; OS = overall survival.

Figure 1A shows OS for the entire cohort, showing a 30-day OS estimate of 67.9%. Figure 1B represents OS categorized by outpatients and hospitalized patients (30-d OS 100% versus 61%; *P* = 0.0005), and Figure 1C represents OS categorized by ICU admission, showing a lower 30-day OS in patients admitted to the ICU (70% versus 42%; *P* = 0.001). Figure 1D compares survival based on CURB-65 score at COVID-19 presentation, showing significantly lower 30-day OS in patients with a CURB-65 score of 2 or ≥3 compared with a 0-1 score (40% and 30%, respectively, versus >80%; *P* = 0.000).

**Figure 2.**
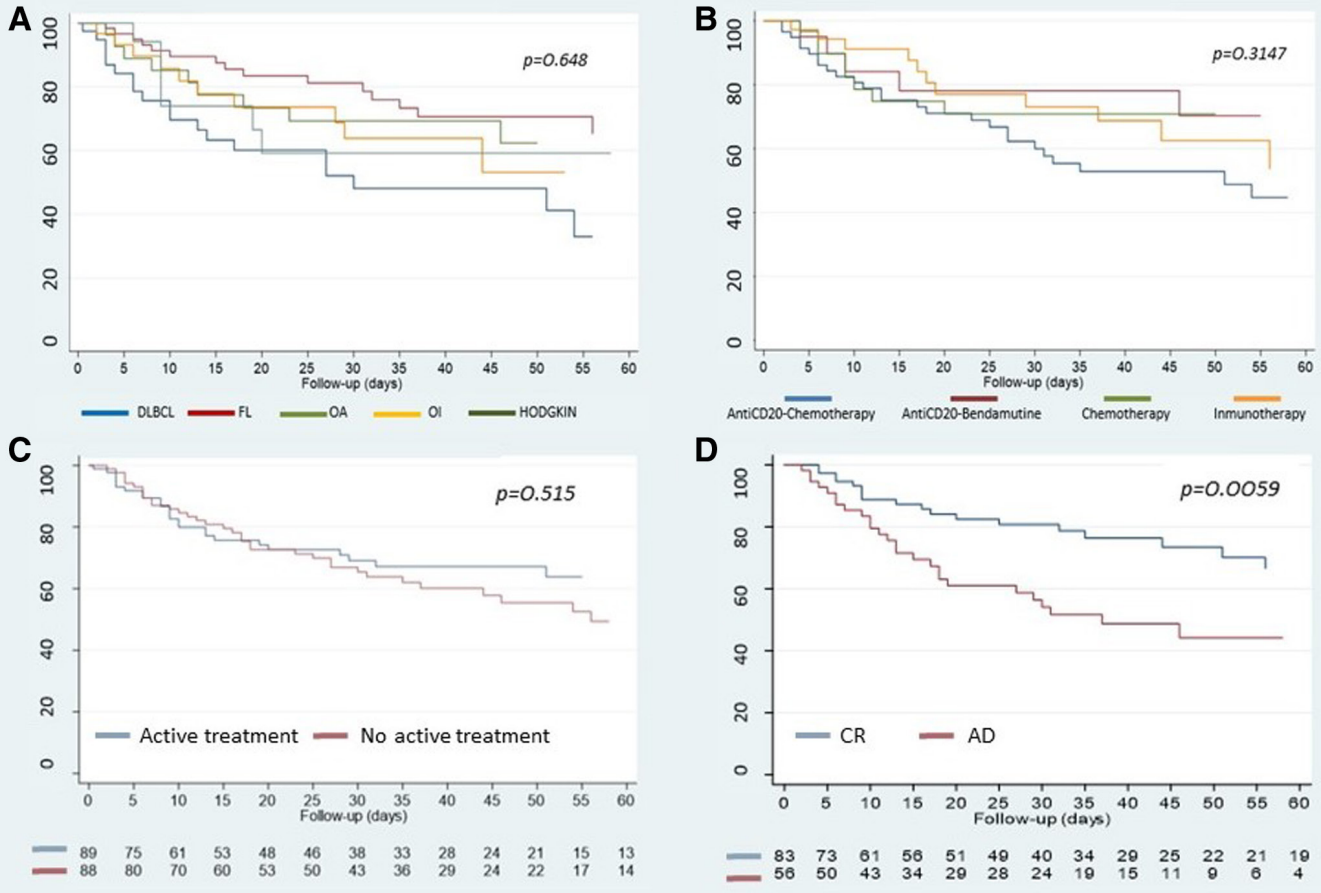
**Overall survival results.** Overall survival categorized by (A) histology subtype, (B) therapeutic regimen, (C) active treatment, and (D) active disease. AD = active disease; CR = complete response; DLBCL = diffuse large B cell lymphoma; FL = follicular lymphoma; OA = other agressive; OI = other indolent.

OS among the various histological types of lymphoma was compared (Figure 2A), showing no differences between them (*P* = 0.0648). However, 30-day OS did significantly differ between DLBCL and FL (50% versus 80%; *P* = 0.0028), showing a poorer outcome in DLBCL.

Figure 2B shows OS categorized according to the therapeutic regimen, showing no differences between the different categories (*P* = 0.155). Of note, regarding the immunotherapy group, only patients with anti-CD-20 therapy were included for this analysis, and the minority groups such as those treated with nivolumab (4 patients) or brentuximab (3 patients) were excluded.

OS was also compared based on active treatment (Figure 2C), without significant differences between the patients who were receiving active treatment and those who were not (*P* = 0.5158).

Figure 2D represents OS in patients with active disease compared with those with complete response, showing a higher survival rate for those with complete response (54% versus 80%; *P* = 0.0059).

## Discussion

This series represents, to our knowledge, the largest series focusing specifically on COVID-19 in the population with lymphoma.

We have described a relatively elderly population with a median age of 70 years, similar to the median age of other cancer series.^4,11,13,17,20,23^ Lymphoma distribution was representative of the general lymphoma population, with FL and DLBCL being the most common subtypes.^30^

Regarding COVID-19 presentation and consistent with other series, symptoms appeared after a median of 5 days (2-7 d),^1,2,4^ with fever and cough as the most frequent.^1-6,14,23^ Dyspnea at presentation was related to mortality.^1-3,14^

Analyzing the effectiveness of the various COVID-19 treatments was not the objective of our study and we have simply described those used. In addition, we now know that many of the drugs used at that time have been discontinued or have proven to be of little efficacy.

More than 85% of our patients required admission, with 9% admitted to the ICU and an overall mortality rate of 34.5%. One of our inclusion criteria was SARS-CoV-2 infection confirmed by RT-PCR. This could have led to an overestimation of the rate of admission and probably the rate of poor outcomes.

The ICU admission rate (9%) was low compared with most of the reported series, ranging from 5% to 38%.^2,4-7,31^ It is worth noting that the study period represents the most severe moment of the pandemic when there was a real limitation in available resources. This overwhelming situation made it necessary to apply more restrictive criteria for ICU admission. Being this a multicenter study, the exclusion criteria for ICU were probably not homogeneous in all cases and this must also be taken into account.

Mortality could also have been influenced by these same conditions, with an overall mortality of 34.5%, high compared with the general large series but similar to the reported mortality in cancer-specific and hematological reports.^3,11,13,23^ In a meta-analysis of hematologic malignancies and COVID-19 that incorporated data from more than 3000 patients, pooled risk of death for lymphomas was 32%,^32^ comparable with the results reported in our series.

In the analysis of mortality predictors, we verified some previously described in nonhematology patients, such as age and the presence of comorbidities such as hypertension, heart disease, and chronic kidney disease.^1,2,4,6,8^ We observed a correlation between a higher CURB-65 score at the time of diagnosis and mortality. d-dimer elevation and lower platelet count, CRP values >10 mg/dL, and LDH >300 U/L were also predictors of death in the univariate analysis. After adjustment, only age >70 years, heart disease, chronic kidney disease, and CURB-65 score ≥2 remained statistically significant mortality predictors.

Among the variables related to lymphoma, the presence of active disease was a predictor of death. However, active treatment, the number of previous lines, or type of treatment did not modify mortality risk.

One of our most remarkable results was finding no differences in survival based on the presence of active treatment (defined in our series as treatment received up to 3 mo earlier). Several studies had found active treatment to be related to poorer outcomes in patients with cancer^7,15^; however, hematology patients represented <15% of the patients in these studies. A larger series in which hematology patients accounted for 22% of the total patients found that treatment in the prior 4 weeks increased the risk of death.^13^ Other studies have not found active treatment to lead to poorer outcomes^11,12,14,23^ and a recent meta-analysis also established a lack of impact of recent treatment on prognosis.^32^

We cannot establish whether active treatment predisposes to more severe infection as virtually all of our patients had an infection severe enough to go to the emergency room and/or be admitted. The conclusion we can draw is that in the population studied, active treatment is not associated with an increased risk of death.

Furthermore, we observed that the presence of active disease (partial response or progression), compared with the complete response situation, increases the risk of mortality. This finding had already been stated in an oncological report^12^ and was also reported in another lymphoma cohort.^33^

The survival analysis confirmed these findings, showing no differences between active and nonactive treatments but reflecting significant differences in OS between active disease and complete response (54% versus 80%; *P* = 0.0059). This finding is relevant due to the utility it can have in clinical practice, given it could be used to support therapeutic decisions. However, it is important to note that the disease situation could have influenced whether a patient was considered eligible for intensive care.

We were not able to demonstrate clear differences between the various lymphoma histologies and therapeutic schemes. It is important to consider that to perform the analyses, these variables were grouped into categories that could have limited the ability to detect factors related to the type of lymphoma and treatment.

DLBCL showed significantly worse OS compared with FL (50% versus 80%; *P* = 0.0028). We performed a subanalysis of this group and found no significant differences regarding the age that could potentially explain the increased mortality rate. However, within the DLBCL dead patients, we found 8 cases of DLBCL new diagnosis, concomitant with COVID-19 diagnosis or in the prior month. We consider mortality could be related to a worse clinical situation of these patients given the characteristics of this lymphoma.

In half of our patients, we were able to obtain serial RT-PCR information for at least 6 weeks after diagnosis. The negativization of RT-PCR has been related to T and B cell counts.^34^ In our series, the criteria for repeating the RT-PCR and the time between them did not follow any protocol, which limits the results. In addition, we did not have semiquantitative available information regarding amplification cycles. Having taken these limitations into account, we interestingly found that there were no significant differences between the types of lymphoma or therapeutic schemes for this setting. However, we observed that in patients with severe clinical course RT-PCR tended to be persistently positive as reported in other recent publications.^35^

Our study has several limitations, such as its retrospective nature and the limited number of included patients. The follow-up time was relatively short and we have focused on lymphoma specific variables.

We have described the reality of patients treated in the early months of the pandemic. A longer follow-up and including a wider spectrum of cases are needed to obtain a more realistic idea of the impact of COVID-19 on this population.

Despite the limitations discussed previously, our results suggest that treatment should not always be delayed or interrupted in these patients, given that those receiving active treatment were shown to not be at higher risk, and achieving disease remission could mean a reduction in mortality risk.

Finally, our data suggest that more severe infection appears to be associated with persistently positive RT-PCR.

## Disclosures

The authors have no conflicts of interest to disclose.

## Sources of funding

This investigation was supported by the grant “Marcos Fernández” from Fundación Leucemia y linfoma.

## Acknowledgments

We thank the Madrid Haematology Association and, especially, Angel Cedillo for the support and all our colleagues for their essential work in bringing these patients forward.

## Supplementary Material


