# Supplementary material for: Risk Factors and Mortality of COVID-19 in Patients With Lymphoma: A Multicenter Study
Source: Hemasphere. 2021 Feb 10;5(3):e538. doi: 10.1097/HS9.0000000000000538 (PMC7886434; doi:10.1097/HS9.0000000000000538)
Supplement: Supplementary file 1 [file hs9-5-e538-s001.docx]

| **Table S1.** Symptoms, radiological and analytical presentation in the full cohort | | | |  |
| --- | --- | --- | --- | --- |
|  | Full cohort (n=177) | Alive  (n=116) | Dead  (n=61) |  |
| **CLINICAL PRESENTATION** | | | | |
| Median days from symptoms onset (IQR) | 5 (2-9) | 5 (5-11) | 4 (2-7) |  |
| Fever (n, %)  Cough (n, %)  Dyspnea (n, %)  Myalgia (n, %)  Diarrhea (n, %)  Chest pain (n, %)  Rhinorrhea (n, %)  Anosmia (n, %)  Odinophagy (n, %) | 134/175 (76.6%)  115/175 (65.7%)  87/175 (49.7%)  45/174 (25.9%)  36/175 (20.6%)  25/175 (14.3%)  15/174 (8.6%)  14/174 (8%)  7/174 (4%) | 86/115 (74.8%)  77/115 (67%)  48/115 (41.7%)  27/115 (23.5%)  26/115 (22.6%)  15/115 (13%)  12/115 (10.4%)  10/115 (8.7%)  7/115 (6.1%) | 48/60 (80%)  38/60 (63.3%)  39/60 (65%)  18/59 (30.5%)  10/60 (16.7%)  10/60 (16.7%)  3/59 (5.1%)  4/59 (6.8%)  2/61 (3.3%) |  |
| CURB-65 (n, %)  0-1  2  ≥3 | 116/175 (66.3%)  29/175 (16.6%)  30/175 (17.1%) | 97/115 (84.3%)  11/115 (9.6%)  7/115 (6.1%) | 19/60 (31.7%)  18/60 (30%)  23/60 (38.3%) |  |
| Radiological findings (n, %)  Normal  Unilateral infiltrate  Bilateral infiltrate  Pleural effusion (n, %) | 18/128 (14.1%)  20/128 (15.6%)  90/128 (70.3%)  15/172 (8.7%) | 14/84 (16.7%)  15/84 (17.9%)  55/84 (65.5%)  5/112 (4.5%) | 4/44 (9.1%)  5/44 (11.4%)  35/44 (79.5%)  10/60 (16.7%) |  |
| **LABORATORY FINDINGS**  Median (IQR) | | | | Reference ranges |
| Leucocyte count (cells/μL)  Neutrophil count (cells/μL)  Lymphocyte count (cells/μL)  Platelets (x10^9^/L)  Haemoglobin (g/dL)  LDH (U/L)  LDH > 225U/L (n, %)  CRP(mg/dL)  CRP >0.5mg/dL (n, %)  Ferritin (μg/L)  Interleukin-6 (pg/mL)  D-Dimer (xULN)  D-Dimer (n, %)  Normal  1-2 ULN  2-4 ULN  4-6 ULN  >6 ULN | 5.1 (3.4-7.2)  3.5 (2.2-5.4)  0.7 (0.3-1.1)  188 (121-268)  12.4 (10.7-13.8)  314 (235-436)  122/157 (77.7%)  7.29 (2.5-16.4)  157/167 (94%)  837 (380-1924)  32.1 (13.3-73.4)  2.2 (1.1-4.4)  28/127 (22%)  30/127 (23.6%)  37/127 (29.1%)  11/127 (8.7%)  21/127 (16.5%) | 5.3 (4.2-7.2)  3.7 (2.4-5.8)  0.7 (0.4-1.2)  199 (135-292)  12.8 (11.3-14.2)  292 (213-383)  72/101 (71.3%)  5.3 (1.8-13.9)  99/109 (90.8%)  769 (387-1690)  29.6 (11.1-71.2)  2.2 (1-3.8)  20/80 (25%)  19/80 (23.8%)  24/80 (30%)  8/80 (10%)  9/80 (11.3%) | 4.2 (2.5-7.4)  3.1 (1.5-5.1)  0.4 (0.3-1)  159 (100-235)  11.6 (10.3-13.1)  395 (283-626)  50/56 (89.3%)  11.7 (5.4-23.2)  58/58 (100%)  1350 (248-2166)  37.4 (16.7-97.6)  2.2 (1.4-7.2)  8/47 (17%)  11/47 (23.4%)  13/47 (27.7%)  3/47 (6.4%)  12/47 (25.5%) | 4 - 10  1.8 – 7.5  1.3 – 3.5  140 - 400  12 - 16  135 - 225  0 – 0.5  5 - 204  <30 |

LDH: lactate dehydrogenase, CRP; C reactive protein

| **Table S2.** COVID-19 management | |
| --- | --- |
| Antiviral treatment (n, %)  Lopinavir/ritonavir  Hydroxychloroquine  Interferon  Azithromycin  Remdesivir  Hyperimmune plasma  Anti-inflammatory treatment (n, %)  Tocilizumab  Anakinra  Corticosteroids  -Methylprednisolone  -Dexamethasone | 89 (50.3%)  156 (88.1%)  13 (7.3%)  79 (44.6%)  9 (5.1%)  7 (4%)  51 (28.8%)  11 (6.3%)  87 (49.2%)  65/87 (74.7%)  20/87 (22.3%) |
| Oxygen requirements (n, %)  Not required  Nasal cannula ≤ 6lpm  Nasal cannula ≥ 6lpm  Reservoir mask  High flow nasal cannula  Tracheal intubation | 46/171 (26.9%)  40/171 (23.4%)  14/171 (8.2%)  51/171 (29.8%)  9/171 (5.3%)  11/171 (6.4%) |

|  | ICU admitted (n=16) | Non eligible (n=51) |
| --- | --- | --- |
| Male/female (%) | 50/50 | 54.9/45.1 |
| Median age (IQR)  Age (n, %)  <60 years  61-74  75-85  >85 | 62 (49-68)  6 (37.5%)  9 (56.3%)  1 (6.3%)  0 (0%) | 77 (73-82)  4 (7.8%)  13 (25.5%)  30 (58.8%)  4 (7.8%) |
| Comorbidities (median, IQR) | 1 (0-2) | 2 (1-3) |
| Histology  DLBCL  Follicular lymphoma  Other aggressive lymphoma^a^  Other indolent lymphoma^b^  Hodgkin lymphoma | 4 (25%)  5 (31.3%)  1 (6.3%)  2 (12.5%)  4 (25%) | 17 (33.3%)  13 (25.5%)  8 (15.7%)  9 (17.6%)  4 (7.8%) |
| Active treatment^c^ (n, %) | 10 (62.5%) | 28 (54.9%) |
| Therapeutics  CD20-chemotherapy  CD20-Bendamustine  Chemotherapy  Molecular targets  Immunotherapy | 6 (37.5%)  1 (6.3%)  3 (18.8%)  1 (6.3%)  5 (31.3%) | 22/43 (51.2%)  5/43 (11.6%)  7/43 (16.3%)  0/43 (0%)  9/43 (20.9%) |
| Response (n, %)  CR  PR  Progression  Not valuable | 13 (81.3%)  3 (18.8%)  0 (0%)  0 (0%) | 14/50 (28%)  7/50 (14%)  15/50 (30%)  14/50 (28%) |
| Active disease^d^ | 3 (18.8%) | 22/36 (61.1%) |
| DLBCL: diffuse large B-cell lymphoma; CR: complete response; PR: partial response  ^a^ Other aggressive lymphomas include mantle cell lymphoma, Burkitt lymphoma and T-cell lymphomas; ^b^ Other indolent lymphomas include marginal zone lymphoma, lymphoplasmacytic lymphoma and hairy cell leukaemia; ^c^ Lymphoma treatment within the previous 3 months; ^d^ Active disease: partial response or progression | | |

**Table S3.** Characteristics of patients admitted to the ICU (n=16) and patients considered non-eligible (n=51).

| **Table S4.** Characteristics of deceased patients (n=61) | |
| --- | --- |
| Male (n, %) | 34 (55.7) |
| Median age (IQR) | 75 (68.5-82) |
| Comorbidities (median, IQR)  Comorbidities (n, %)  None  1-2  3-4  >4 | 2 (1-3)  9 (14.8%)  37 (60.7%)  12 (19.7%)  3 (4.9%) |
| Histology  DLBCL  Follicular lymphoma  Other agressive lymphoma^a^  Other indolent lymphoma^b^  Hodgkin lymphoma | 19 (31.1%)  15 (24.6%)  10 (16.4%)  10 (16.4%)  7 (11.5%) |
| Active treatment^c^ (n, %) | 34 (55.7%) |
| Lines of treatment  0  1  2  3  ≥4 | 7/59 (11.9%)  33/59 (55,9%)  11/59 (18,6%)  5/59 (8,5%)  3/59 (5,1%) |
| Therapeutics  CD20-chemotherapy  CD20-Bendamustine  Chemotherapy  Molecular targets  Immunotherapy | 27 (50,9%)  5 (9,4%)  8 (15,1%)  1 (1,9%)  12 (22,6%) |
| Response (n, %)  CR  PR  Progression  Not evaluated | 20/60 (33.3%)  11/60 (23.3%)  15/60 (25%)  14/60 (23.3%) |
| Active disease^d^ | 26/46 (56.5%) |
| DLBCL: diffuse large B-cell lymphoma; CR: complete response; PR: partial response  ^a^Other aggressive lymphomas include mantle cell lymphoma, Burkitt lymphoma and T-cell lymphomas; ^b^Other indolent lymphomas include marginal zone lymphoma, lymphoplasmacytic lymphoma and hairy cell leukaemia; ^c^ Lymphoma treatment within the previous 3 months; ^d^ Active disease: partial response or progression | |
